# Generating Robust and Informative Nonclinical *In Vitro* and *In Vivo* Bacterial Infection Model Efficacy Data To Support Translation to Humans

**DOI:** 10.1128/AAC.02307-18

**Published:** 2019-04-25

**Authors:** Jürgen B. Bulitta, William W. Hope, Ann E. Eakin, Tina Guina, Vincent H. Tam, Arnold Louie, George L. Drusano, Jennifer L. Hoover

**Affiliations:** a Center for Pharmacometrics and Systems Pharmacology, Department of Pharmaceutics, College of Pharmacy, University of Florida, Orlando, Florida, USA; b Centre for Antimicrobial Pharmacodynamics, Department of Molecular and Clinical Pharmacology, Institute of Translational Medicine, University of Liverpool, Liverpool, United Kingdom; c National Institute of Allergy and Infectious Diseases, National Institutes of Health, Rockville, Maryland, USA; d College of Pharmacy, University of Houston, Houston, Texas, USA; e Institute for Therapeutic Innovation and Department of Medicine, College of Medicine, University of Florida, Orlando, Florida, USA; f Antibacterial Discovery Performance Unit, GlaxoSmithKline, Collegeville, Pennsylvania, USA

**Keywords:** best practices, drug development, hollow fiber system, *in vitro* infection models, mouse infection models, optimal design, pharmacokinetics/pharmacodynamics, progression and decision criteria, validation, workshop summary

## Abstract

In June 2017, the National Institute of Allergy and Infectious Diseases, part of the National Institutes of Health, organized a workshop entitled “Pharmacokinetics-Pharmacodynamics (PK/PD) for Development of Therapeutics against Bacterial Pathogens.” The aims were to discuss details of various PK/PD models and identify sound practices for deriving and utilizing PK/PD relationships to design optimal dosage regimens for patients. Workshop participants encompassed individuals from academia, industry, and government, including the United States Food and Drug Administration. This and the accompanying review on clinical PK/PD summarize the workshop discussions and recommendations. Nonclinical PK/PD models play a critical role in designing human dosage regimens and are essential tools for drug development. These include *in vitro* and *in vivo* efficacy models that provide valuable and complementary information for dose selection and translation from the laboratory to human. It is crucial that studies be designed, conducted, and interpreted appropriately. For antibacterial PK/PD, extensive published data and expertise are available. These have been leveraged to develop recommendations, identify common pitfalls, and describe the applications, strengths, and limitations of various nonclinical infection models and translational approaches. Despite these robust tools and published guidance, characterizing nonclinical PK/PD relationships may not be straightforward, especially for a new drug or new class. Antimicrobial PK/PD is an evolving discipline that needs to adapt to future research and development needs. Open communication between academia, pharmaceutical industry, government, and regulatory bodies is essential to share perspectives and collectively solve future challenges.

## Abstract (precis)

In June 2017, the National Institute of Allergy and Infectious Diseases, part of the National Institutes of Health, organized a workshop entitled “Pharmacokinetics-Pharmacodynamics (PK/PD) for Development of Therapeutics against Bacterial Pathogens.” The aims were to discuss details of various PK/PD models and identify sound practices for deriving and utilizing PK/PD relationships to design optimal dosage regimens for patients. Workshop participants encompassed individuals from academia, industry, and government, including the United States Food and Drug Administration.

## INTRODUCTION

Nonclinical infection models are commonly used to characterize pharmacokinetic/pharmacodynamic (PK/PD) relationships for antibacterials and provide critical information for designing human dosage regimens (1). The discipline of PK/PD has been developing for several decades, and there is extensive evidence demonstrating that nonclinical infection models can predict clinical outcomes (1, 2). Since typical antibacterial drugs target the pathogen and not the host, the basic antimicrobial pharmacology and microbiology of the drug-pathogen interaction can be studied outside the clinical setting. These insights can be assumed to hold true, in general, for drug-pathogen interactions that occur during infection of a human host (3). While there are many elements that cannot easily be studied outside the setting of a human infection, the insights gained from nonclinical infection models strongly support the rational design of optimal antibacterial dosage regimens for evaluation in future clinical trials.

The goal of conducting nonclinical PK/PD infection models is, first and foremost, to elucidate exposure-response relationships and to subsequently design and optimize dosage regimens. It is crucial to understand how drug concentration profiles at the primary infection site can maximize bacterial killing and minimize the emergence of bacterial resistance. Armed with this knowledge, dosage regimens can be designed to balance these goals while maintaining an acceptable level of safety in humans. Establishing exposure-toxicity relationships and identifying optimal regimens which account for between-patient variability can greatly support achieving this balance (4, 5).

The existing armamentarium of PK/PD models is commonly employed to support these goals throughout the phases of drug development. Data from nonclinical PK/PD models are indispensable for selecting the doses and regimens for patients, establishing susceptibility breakpoints, and ultimately refining clinical dosage regimens. The latter should reliably achieve PK/PD targets to maximize the probability that all patients will achieve efficacious drug exposures while limiting resistance development.

In the current environment, it can be challenging or virtually impossible to find and recruit a sufficient number of patients (e.g., those with infections caused by multidrug-resistant pathogens) for multiple, large-scale clinical trials designed for inferential testing. Consequently, there may be a heavy reliance on nonclinical PK/PD data to support and enhance the insights gained from human studies. These data also comprise an important element of regulatory submissions, as evidenced by guidelines published by the European Medicines Agency (EMA) (6, 7). For submissions to the Center for Drug Evaluation and Research that rely on limited clinical data, the importance of nonclinical PK/PD information is magnified, and nonclinical data packages need to be thorough to strongly support safety and efficacy in patients (8).

Generating robust nonclinical PK/PD data was a key topic in the workshop sponsored by the National Institute of Allergy and Infectious Diseases (NIAID) in June 2017 entitled “Pharmacokinetics-Pharmacodynamics (PK/PD) for Development of Therapeutics against Bacterial Pathogens.” This review aims to summarize the information presented and discussed regarding nonclinical PK/PD models. Workshop participants came from across academia, industry, and government, including the United States Food and Drug Administration (FDA), to provide a wide range of perspectives. Characterizing PK/PD for new drugs can be complex, and there is no single road map that can be applied for all drugs. In this review, we sought to provide guidance and considerations for designing, performing, and interpreting studies to develop a robust and informative nonclinical PK/PD package. Moreover, we aimed to put the roles of these models into perspective to design safe and effective dosage regimens for future clinical studies.

## *IN VITRO* PK/PD MODELS

Static concentration time-kill (SCTK) assays are suitable screening tools for assessing drug structure activity and exposure-response relationships and for choosing informative drug exposures for subsequent dynamic infection model studies over longer treatment durations. SCTK studies are used to assess antibacterial activity and are typically performed over 24 (to 48) h. They use constant antibiotic concentrations and assume no or limited drug degradation; however, this should be experimentally confirmed, especially in studies with resistant strains. This experimental model can efficiently assess exposure-response relationships against the predominant bacterial population for antibiotic monotherapy and evaluate PD drug interactions for combinations. Further, SCTK studies can identify the rate of bacterial killing, help to define whether microbial killing is concentration or time dependent, and identify antibiotic exposures that maximize bacterial killing and minimize regrowth, as well as evaluate the effect of the initial bacterial inoculum on antibiotic activity (9–11). Depending on the study objectives, viable counts on agar plates with and without the antibiotic can be utilized to determine the impact of drug exposure on both total and less-susceptible bacterial populations and identify whether regrowth is caused by less-susceptible bacteria (12–14). Results from 24-h or 48-h SCTK studies may predict outcomes in the dynamic one-compartment model (chemostat) or hollow fiber infection model (HFIM) for the first 24 to 48 h but not at later time points.

The SCTK can efficiently assess a large number of treatment and control arms. Other advantages include its low cost and minimal equipment requirements; limitations include the use of constant drug concentrations and typically short treatment duration (24 to 48 h). The study duration can be extended to over 1 week, if needed, by replacing the medium with fresh (antibiotic-containing) broth every 24 h. For less-stable drugs, small antibiotic doses can additionally be supplemented to offset degradation (15). Dynamic *in vitro* PK/PD models offer the additional capability of evaluating the effect of drug concentrations that change over time and can thereby mimic drug concentration profiles in humans. Dynamic systems include the one-compartment model (also called chemostat) and the two-compartment HFIM (16–20). To more precisely achieve PK/PD targets in these more labor-intensive dynamic infection models, it is often beneficial to perform arithmetic MIC determinations using finer-than-2-fold dilutions, particularly for higher MIC values (e.g., >0.5 mg/liter) where the large incremental increases in test concentrations reduce the precision of the measurement (e.g., lower test concentrations have 2 to 3 significant figures, while higher test concentrations are reported with only 1 significant figure).

### Dynamic one-compartment models.

**FIG 1:**
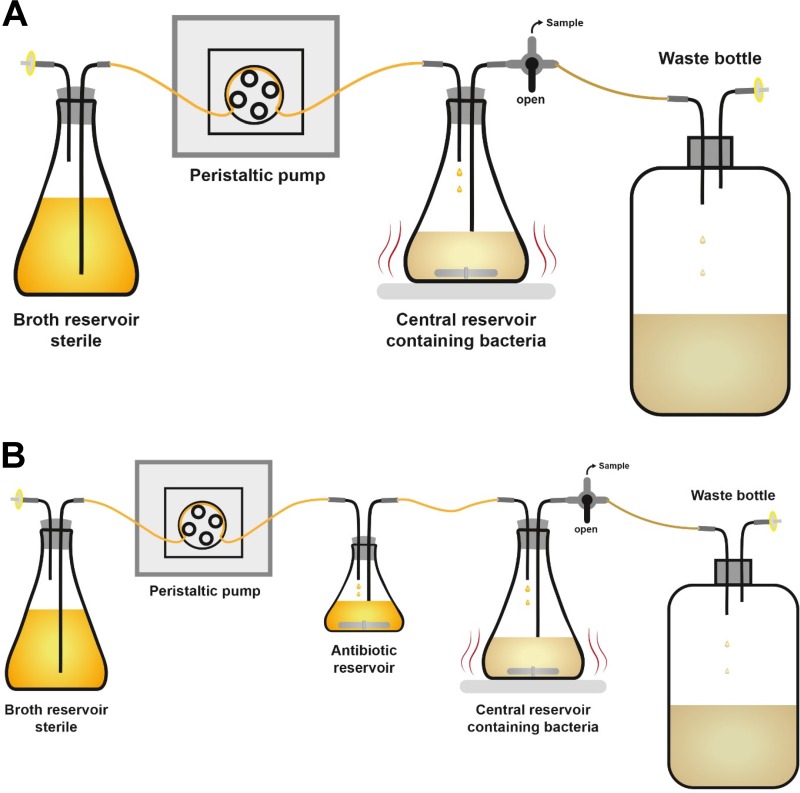
Dynamic one-compartment *in vitro* infection model (“chemostat”). Fresh medium is added continuously while culture contents are removed at the same rate to maintain a constant volume. (A) Chemostat model for simulating a monoexponential decline of drug concentrations after intravenous dosing; the antibiotic(s) is/are dosed into the central reservoir as bolus doses or zero-order infusions. (B) Chemostat for oral dosing, which can simulate drug concentration-time profiles with first-order absorption and elimination; typically, the antibiotic(s) is/are dosed into the antibiotic reservoir as bolus doses.

Chemostats are one-compartment, bacterial culture bioreactors with a typical culture volume of 100 to 250 ml. Fresh medium is added continuously while culture contents are removed at the same rate to maintain a constant volume (16). Drugs are either administered directly as a bolus or infused (via a pump) into the bioreactor or provided as a continuous infusion with the inflowing medium (Fig. 1). The chemostat can simulate drug concentrations changing over time following a single half-life to evaluate efficacy. This model can also assess dose fractionation by splitting the same daily dose into various dosing intervals. Moreover, chemostats can simulate different durations of infusion and front-loaded regimens, for example (Table 1) (21–24). With the continuous replenishment of growth medium and nutrients, the one-compartment system supports testing longer treatment durations for dose-range and dose fractionation studies. This system can simulate the time course of antibiotic concentrations for monotherapies and combinations to study bacterial killing and regrowth.

**TABLE 1:** Types of experiments that can be performed with widely used nonclinical PD infection models^*a*^

Study objective	Static time-kill model	One-compartment system (“chemostat”)	Two-compartment hollow fiber system	Mouse infection model
1. Dose-range study: killing of predominant population	Yes^*b*^	Yes^*b*^	Yes^*b*^	Yes^*b*^
2. Dose-range study: suppression of resistance	±^*c*^	±^*c*^	Yes^*c*^	±^*c*^
3. Dose-fractionation study: killing of predominant population	No	Yes	Yes	Yes
4. Dose-fractionation study: suppression of resistance	No	±	Yes	±
5. Combination therapy: killing of predominant population	Yes	Yes (short term)	Yes	Yes
6. Combination therapy: suppression of resistance	±	±	Yes	±
7. Toxin suppression by drugs	Yes	±	Yes	Yes
8. Dissecting the interaction of the parent drug and metabolites on antimicrobial effect	±^*d*^	±^*d*^	Yes^*d*^	No
9. Effect of physiological state of bacteria on drug activity	±	±	Yes	±
10. PD index for drug toxicity	No	No (unless toxicity is acute)	Yes	±^*e*^

a PD, pharmacodynamic; ±, study objective can potentially be addressed in this system.

b Bacterial strains which display the lowest mutation frequency of resistance should be avoided in dose-range studies; instead, strains which best represent the most commonly observed mutation frequencies are preferred.

c Strains with a relevant resistance mechanism(s) should be chosen for *in vitro* studies. The MIC_50_ and MIC_90_ for the pathogen of interest may be used to guide strain selection.

d A biologically active metabolite(s) needs to be available, since it is most likely not formed in the *in vitro* system.

e Some dosage regimens (e.g., those used to assess time over a toxicity threshold) may also lead to high peak concentrations, especially for short-half-life drugs, which complicates the interpretation of these studies.

Limitations of the chemostat include the potential for washout of bacteria and contamination of the medium, particularly for studies with longer treatment duration. Most published studies have been conducted over 96-h or shorter durations (and often over only 24 h). Simulating concentration-time profiles for drugs with a short half-life in the chemostat results in washout of a considerable number of bacteria. The latter causes the drug exposure needed for bacterial killing and resistance prevention to be underestimated, especially for slowly replicating bacteria or subpopulations. Filters can be used to help mitigate this issue but are not ideal due to clogging by bacteria (20, 25). Both washout of bacteria and incomplete oxygenation can lead to substantially lower maximum bacterial densities in the chemostat than in SCTK and HFIM. Depending on the simulated half-life, bacterial waste products may accumulate over time in the chemostat. These features limit the ability of the chemostat to evaluate bacterial killing and resistance prevention at high bacterial densities and over long study durations.

### Dynamic two-compartment models.

**FIG 2:**
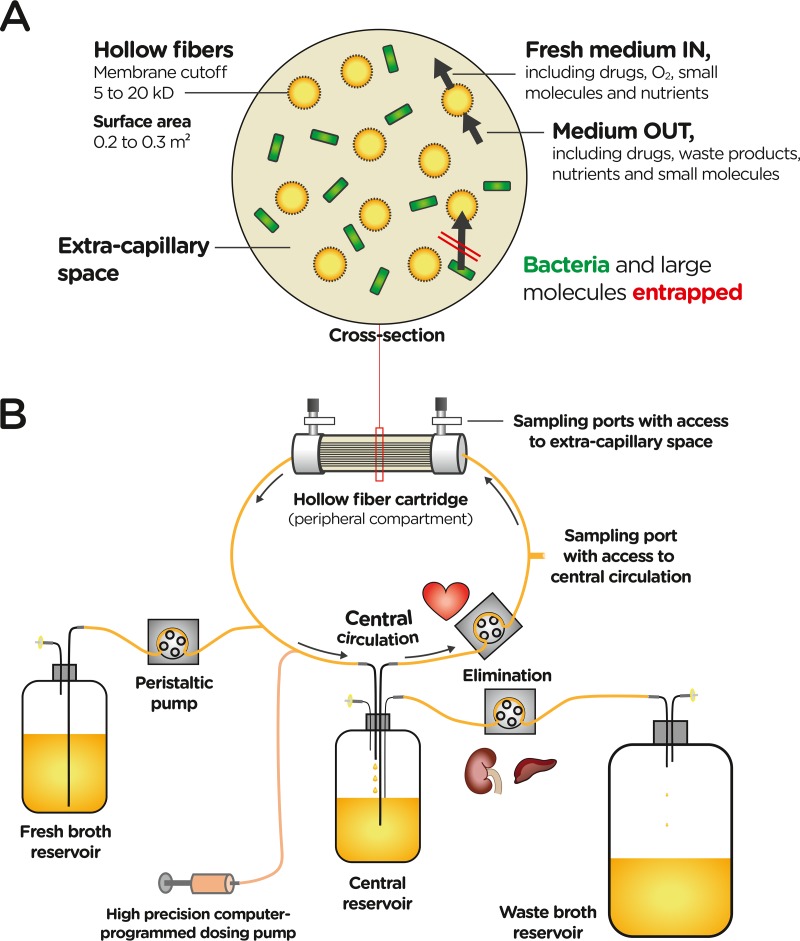
Dynamic two-compartment hollow fiber *in vitro* infection model. (A) Cross section of a hollow fiber cartridge. Many hollow fibers provide a large surface area (typically 0.2 to 0.3 m^2^, depending on the cartridge). According to the molecular weight cutoff of the hollow fiber membrane, medium, drugs, oxygen, nutrients, bacterial metabolites (“waste products”), and other small molecules can exchange between the central circulation (which includes the interior of the hollow fibers) and the extracapillary space of the cartridge. In contrast, bacteria, other cells (if present), and large molecules are entrapped in the extracapillary space of the hollow fiber cartridge. (B) Flow of broth medium from the fresh broth to the central reservoir. From the latter, broth is circulated to the peripheral compartment (i.e., the extracapillary space of the hollow fiber cartridge) or is eliminated. Elimination occurs from the central reservoir into the waste broth reservoir. A high-precision dosing pump is used to dose drugs into the central circulation.

In our opinion, the HFIM is the preferred and most capable *in vitro* model for evaluating PK/PD indices (26) and concentrations that best predict bacterial killing and resistance prevention (Table 1). The HFIM is a two-compartment system where bacteria are entrapped in the extracapillary space of a hollow fiber cartridge that serves as a peripheral infection site (Fig. 2). This system can simulate virtually any time course of drug concentrations for one or multiple drugs with the same or different half-lives (27–30). Multiexponential profiles can be simulated by switching the pump rates at appropriate times (31). Bacteria are contained within the peripheral compartment of the hollow fiber cartridge, which completely prevents washout of bacteria. The cartridge has a large surface-to-volume ratio (32), providing optimized growth conditions for aerobic bacteria since bacteria are constantly exposed to fresh broth and oxygen, and waste products are continually removed (Fig. 2). Thus, the maximum achievable bacterial density is usually over 1 order of magnitude higher in the HFIM than in the SCTK assay. Due to these differences in growth conditions, the SCTK model tends to show extensively attenuated bacterial killing at a high compared to low initial inocula for some drug classes (9, 10, 33). This attenuation (i.e., an inoculum effect) tends to be less pronounced in the HFIM (34, 35), since bacterial replication is faster in the HFIM than in the SCTK model at the same bacterial density (e.g., 10^8^ CFU/ml). The clinical relevance of experimental inoculum effects is not fully understood; however, it has been shown in a mouse model that higher drug exposures are required to achieve stasis or 1-log_10_ killing against a higher (10^7^ CFU/ml) than a lower (10^5^ CFU/ml) inoculum of multiple Staphylococcus aureus strains for four classes of antibiotics (36).

The HFIM offers the advantage that it can assess resistance prevention over typical antibiotic treatment durations for serious bacterial infections in patients (i.e., approximately 5 to 14 days). For slowly replicating bacteria such as Mycobacterium tuberculosis, studies can be extended to 28 days (37, 38) and longer, if needed. Moreover, the HFIM is the most capable and informative *in vitro* model for evaluating the efficacy of drug combination regimens and front-loaded dosage regimens and of antibiotics with a short half-life, since there is no washout of the microbe (39, 40). The HFIM is further suitable for studies with highly communicable or virulent biosafety level 3 (BSL3) pathogens (such as Mycobacterium tuberculosis, Bacillus anthracis, Burkholderia mallei, Burkholderia pseudomallei, Francisella tularensis, and Yersinia pestis), since the bacteria are contained in the HFIM cartridge (27, 41).

Limitations of the HFIM include its relatively high cost, which is compounded by the single use of cartridges, and the more extensive effort required to plan, set up, and execute studies. Some (lipophilic) drugs bind to HFIM components, which hinders their testing. Different hollow fiber materials (including cellulosic, polysulfone, and polyvinylidene difluoride [PVDF]) are available to minimize binding, if needed (32). Given the molecular weight cutoff of HFIM cartridges, β-lactamase enzymes are entrapped in the extracellular space. For subtherapeutic regimens which provide limited or no bacterial killing, β-lactamase enzymes may accumulate over time in the cartridge and degrade β-lactams (35). This is likely moderated by bacterial proteases that break down β-lactamase enzymes and can be mitigated by washing of the bacterial suspension before it is inoculated into the HFIM cartridge. Therefore, quantifying β-lactam concentrations in the extracapillary space of the HFIM cartridge (Fig. 2) is warranted for β-lactamase (over)-producing strains. This is also essential for high-inoculum studies of resistant strains for highly permeable pathogens such as Escherichia coli. These β-lactamase-producing strains can cause a rapid decline of the extracellular β-lactam concentration due to β-lactamase activity in the periplasmic space of bacteria, an issue which also applies to SCTK and chemostat studies.

## CONSIDERATIONS FOR DESIGN AND CONDUCT OF *IN VITRO* PK/PD MODELS

### Strain selection.

Robust PK/PD analyses require examination of multiple strains that should include one reference strain (e.g., a widely available ATCC strain) and one susceptible and two less-susceptible clinical isolates; the latter may include one strain from an intensive care unit (ICU) patient and one strain from a non-ICU patient. Strains should be relevant for the clinical indication and study purpose; they should include different resistance mechanisms and a relevant (i.e., wide) range of susceptibility to the studied drug(s). Studies evaluating isogenic sets of strains can provide valuable information about the impact of a specific resistance mechanism.

Furthermore, the chosen strains should represent the most common mutation frequency (MF), and strains with the lowest MF (i.e., strains with a small number of preexisting resistant mutants) should be avoided. This necessitates determining the MF for a range of strains; it is recommended to test at least three strains of a given bacterial species for this purpose. For strains with multiple bacterial populations of different levels of susceptibility to an antibiotic, the impact of these less susceptible populations on PK/PD relationships and targets may need to be evaluated (34, 42). Appropriate reference strains (such as ATCC strains) should be used throughout the research program to demonstrate reproducibility. Finally, if possible, the chosen strains should be virulent in animal models to support efficient translation to animal studies, and virulence should be confirmed before conducting HFIM studies.

### Inoculum and mutation frequency.

The initial bacterial inoculum needs to be relevant for the clinical indication and study purpose. A high inoculum with a total bacterial burden of approximately 10^8.5^ CFU or greater (equivalent to 15 ml of a bacterial suspension at 10^7.3^ CFU/ml in the HFIM) is typically used in studies that target ventilator-associated and hospital-acquired bacterial pneumonia (VABP/HABP) and in resistance prevention studies (43). Experiments with a total bacterial inoculum lower than approximately 10^6^ CFU (equivalent to 15 ml of a bacterial suspension at 10^4.8^ CFU/ml or lower) are usually not relevant for clinical indications. However, such low inoculum studies may be highly suitable to address mechanistic research questions on the rate of *de novo* formation of resistant mutants or on phenotypic tolerance of the predominant population (in the absence of preexisting mutants at initiation of therapy), for example. Knowing the MF for the tested antibiotic(s) is essential (14). By considering the expected number of resistant mutants in the initial inoculum, one can increase or decrease the probability of a resistant mutant being present or absent, depending on the study objectives. To assess suppression of amplification of preexisting less-susceptible mutants, the number of bacteria in the total system volume should be at least 1 log_10_ CFU higher than the inverse of the MF. This ensures that all treatment and control arms contain at least one preexisting less susceptible mutant (with a probability of 99.9% for a 16-arm study, see useful formulas in the supplemental material).

### Duration of therapy and resistance prevention.

The study duration depends on the study objectives. To determine the PK/PD index (e.g., AUC/MIC [the area under the concentration-time curve over 24 h at steady state divided by the MIC], peak/MIC, or *T*>MIC [cumulative time {in hours} of a 24-h period that the drug concentration exceeds the MIC under steady-state pharmacokinetic conditions]) that best predicts bacterial killing, short-term studies over approximately 1 to 3 days may be sufficient; longer studies are required for slowly replicating bacteria and should consider the cell division time. These data can be used to determine the drug exposure required to achieve a 1-log_10_ or 2-log_10_ reduction in bacterial burden or bacteriostasis at 24 h and end of study. To assess the drug exposure and dosage regimens that suppress resistance amplification, the treatment duration should mimic the therapy duration for the intended clinical indication (usually at least 5 to 8 days). Some antibiotic classes show emergence of resistance more rapidly (21, 34), but absence of resistance emergence over the first 2 days often does not correlate with resistance prevention over 10 days. Therefore, HFIM studies to evaluate resistance prevention often use 7, 10, or 14 days of treatment (28, 44).

### Drug stability.

It is critical to evaluate drug solubility and stability under relevant conditions (e.g., solvents, media, storage, and experiment temperatures, as well as durations consistent with those of the planned experiments) (45, 46). Many antibiotics are hydrophilic and soluble in water (47), but some have limited solubility and their concentrations may decrease over time due to (slow) precipitation. In addition, drugs may bind nonspecifically to flasks, tubing, filters, and fibers; thus, it is important to assess whether these issues exist for the drug(s) to be studied.

### Drug concentration profiles.

When available, protein binding and pharmacokinetic data from patients with an infection should be used to simulate the non-protein-bound (or “free” [*f*]) concentration-time course of drugs in plasma or, ideally, tissue exposures at the primary infection site for the intended clinical indication (e.g., lung epithelial lining fluid [ELF] for pneumonia). This is important because exposure profiles in patients may differ from those in healthy volunteers, and between-patient variability in PK can be substantial in the critically ill. Of note, infection and the associated inflammation can alter drug exposure in ELF or cerebrospinal fluid (CSF) (48, 49) and some antibiotics have heterogeneous distribution across major tissues and organs. For example, polymyxin B accumulates in kidney (50) but less in lung (51). It is further important to understand and simulate non-protein-bound (i.e., free) drug exposures that are relevant to the infection site.

If an active metabolite contributes to the overall bacterial killing, the parent and metabolite should be evaluated separately and the concentration-time profiles of both compounds should be generated *in vitro* at the values found at the intended infection site in patients. This provides the most accurate characterization of bacterial killing and resistance prevention for antibiotics with an active metabolite. For prodrugs that are inactive and/or rapidly converted to the parent, such as tedizolid, ceftaroline, or colistin methanesulfonate, the drug exposure and PK profile of the biologically active compound should be dosed in *in vitro* PD systems (52, 53) due to different formation rates *in vitro* and *in vivo*.

### Quantifying drug concentrations.

Determining the time course of achieved drug concentrations in dynamic PK/PD models is a best practice, both to validate the simulated PK profiles and to provide observed data for analysis. This is an essential step, rather than relying solely on mathematically predicting the expected drug exposures. This is particularly important for intermittent dosing and complex dosage regimens (e.g., front loading [40, 54]). Collecting these data allows correlation of actual drug exposures with the extent of bacterial killing and resistance suppression and may explain unexpected results.

Drug concentrations should be quantified using multiple times per dosing interval, e.g., at approximately 30 min after the end of infusion (to allow for proper equilibration of the system), one to three intermediate samples, and a sample toward the end of the dosing interval. This sampling scheme should be adjusted for more-complex regimens and repeated during multiple dosing intervals to confirm reliability of the dosing (including the syringe pump) and performance of the peristaltic pump and to characterize attainment of steady state (35).

### Quantifying bacterial populations.

The impact of drug exposure on the total and less-susceptible bacterial populations should be assessed (12, 13, 27–31, 34) when the study objectives include assessing resistance prevention. The importance of conducting these types of studies is described in the supplemental material. Killing of the predominant bacterial population is usually determined by quantitative viable counts on antibiotic-free agar. In contrast, killing and amplification of the less-susceptible bacterial population(s) are assessed by viable counting onto antibiotic-containing agar. Subculturing should be done on agar containing the same antibiotic(s) used in a respective treatment arm and on agar containing all antibiotics for the growth control. Agar containing 3× and 5× the MIC is commonly used; however, this choice depends on the initial (i.e., pretreatment) MIC and the step size of the MIC change (e.g., due to loss of an outer membrane porin [OprD] or upregulation of an efflux pump) associated with a relevant resistance mechanism(s). The MF can also guide selection of an appropriate antibiotic concentration(s) in agar that should be between the MIC of the parent strain and that of the first-step mutant. To identify potential second- and third-step mutants with further decreased susceptibility, higher multiples of the MIC in agar can be used. For drugs with a large MIC increase in first-step mutants, higher multiples of the MIC or a fixed concentration in agar (e.g., 300 mg/liter rifampin for Pseudomonas aeruginosa) can be employed (55). Strains with high baseline MICs and combination therapy studies require special attention for selecting the most suitable antibiotic concentrations in agar to quantify the less susceptible population(s) (56).

For most antibiotics, enumerating colonies of subcultured bacteria after 24 h of incubation on antibiotic-containing agar is not sufficient and may greatly underestimate the less-susceptible population. Additional colonies may become visible after 48 to 72 h of incubation. Loss of moisture in agar can be minimized via the use of a humidified incubator or of an increased agar volume per plate or by incubating a tray of agar plates in a partially opened plastic bag. Drug stability in the agar during incubation should be experimentally tested, especially for “bacteriostatic” antibiotics that inhibit growth but cause only slow bacterial killing. Moreover, the MICs should be determined for a subset of colonies growing on antibiotic-containing agar to validate their decreased susceptibility to the antibiotic.

### Data analysis approaches.

Empirical and mechanism-based (MB) mathematical models both have their roles for analyzing *in vitro* PK/PD data. Empirical models (23, 57–72) are efficient and typically analyze viable counts at the end of therapy or the area under the viable count curve (on linear or log scale) during different time intervals (e.g., from 0 to 5 h, 0 to 24 h, and 0 h to end of study). Time-independent exposure-response relationships can identify exposure targets for efficacy and empirically describe the observed synergy of drug combinations; however, time-independent exposure-response analyses are not suitable to rationally optimize combinations or monotherapy regimens with changing dose intensity over time (e.g., front loading) and do not describe the time course of drug concentrations. Empirical time course models can describe drug concentration and viable count profiles but lack mechanistic insights (e.g., receptors) and do not account for multiple resistance mechanisms. Particularly for combination therapy, empirical models cannot rationally optimize the effects elicited by antibiotics with multiple target sites or multiple mechanisms of action (10, 73, 74) or for combinations with several synergy mechanisms (14, 35, 75).

Mechanism-based (MB) as well as quantitative and systems pharmacology (QSP) models have been developed to overcome many of these limitations. While MB and QSP models both implement the mechanism(s) of drug action or mechanism(s) of resistance or both, QSP models usually describe multiple different types of experimental observations to characterize these mechanisms in more depth. Both of these models can simultaneously describe and predict the time course of bacterial killing and resistance emergence, and both have been developed for antibiotic monotherapy and combinations (9, 10, 14, 15, 30, 31, 33, 34, 40, 41, 55, 56, 58, 71, 72, 76–84). These models incorporate genotypic resistance development by multiple bacterial populations with different susceptibilities and phenotypic tolerance of slowly replicating bacteria. They offer the advantage of integrating molecular experimental data and allow rational optimization of innovative monotherapy and combination dosage regimens (including front loading) for more than two drugs, if needed. Further, translational MB and QSP models can incorporate toxicodynamics (4, 5, 39, 85, 86) and account for the impact of the immune system (87–90). Independently of the approach employed, prospective experimental validation is essential (31, 72).

### Interpretation of results.

In interpreting *in vitro* PK/PD results, it is important to consider the mode of drug action, i.e., whether the antibiotic is rapidly or slowly killing and which endpoint (e.g., stasis or 1-log_10_ or 2-log_10_ killing) is most clinically relevant. A stasis endpoint may be sufficient for some less acute clinical indications such as uncomplicated skin and skin structure infections and complicated urinary tract infections (cUTI). However, 1- or 2-log_10_ killing may be more desirable for severe infections (such as VABP). In addition, while the primary PK/PD index is often consistent between different pathogens and strains, the drug exposures required to achieve a target endpoint may vary greatly (91). This may have implications for translation to broad coverage and clinical utility of antibiotics (53, 92–94). Moreover, this reinforces the need to include a sufficiently diverse spectrum of bacterial strains in nonclinical PK/PD models and to consider the potentially substantial between-patient variability in PK, especially in unstable patients with sepsis or septic shock (see companion review [95]).

Potential extreme observations that fall outside a predetermined threshold for an “outlier” (e.g., >2 standard deviations [SD] from the mean) should not be automatically discarded. Such a data point(s) may represent an unexpected but important behavior (e.g., a mutation, with low frequency, leading to emergence of resistance or to development of tolerance to the drug). While mathematical approaches are available to handle potential “outliers,” experimental replicates and further laboratory investigation (such as characterization of resistant mutants and/or evaluation of potential drug tolerance) are strongly preferred.

## CHALLENGES OF INTERPRETING *IN VITRO* RESULTS

The data generated using *in vitro* systems provide valuable insights into the direct interaction between the pathogen and the drug, and it is recommended that drug developers incorporate these types of models into their development programs. However, in some cases, the results may not directly translate to the clinic because *in vitro* systems do not fully mimic the *in vivo* environment. The PK/PD targets required in patients may be lower or higher than those *in vitro* if host factors affect bacterial killing or if the fitness of resistant mutants is reduced *in vivo* (96). An *in vivo* PK/PD target may be lower if the immune response contributes significantly to bacterial killing (3, 88); conversely, the *in vivo* target may be higher if host factors reduce the susceptibility of the bacteria (e.g., due to binding to lung surfactant or to persistence in deep-seated or sequestered infection sites). Moreover, drug binding in plasma needs to be considered, since generally only free (i.e., unbound) drug is available to interact with bacterial receptors. Therefore, translation of PK/PD targets should be based on free drug concentrations unless another rationale (e.g., for very highly bound drugs) is provided. It should be noted that *in vitro* studies generally do not incorporate plasma proteins (by design). Binding of many antibiotics to the *in vitro* pharmacodynamic systems is negligible (91), and the experiments inherently characterize free drug. This is in contrast to *in vivo* studies, in which results should be adjusted for protein binding in the test species.

For emergence of resistance studies, it may be prudent to interpret results as an assessment of risk in the absence of host factors (e.g., the immune system) rather than as a direct prediction of clinical outcome. For example, while *in vitro* models are excellent for studying aminoglycosides as part of combination regimens (15, 31, 35, 55, 56, 81, 97), they are not suitable for testing aminoglycoside monotherapy because this drug class readily generates small-colony variants that are less common *in vivo* (10, 12, 96, 98). For the bacterial populations that cause failure of therapy *in vitro*, assessing the resistance mechanism(s), the ability of high drug concentrations to kill these mutants, and the MIC shifts toward potential partner antibiotics may be valuable. Further, evaluating synergistic drug combinations, as well as *in vivo* fitness and virulence (99), may guide translation to animal models and ultimately to patients.

## *IN VIVO* PK/PD MODELS

**FIG 3:**
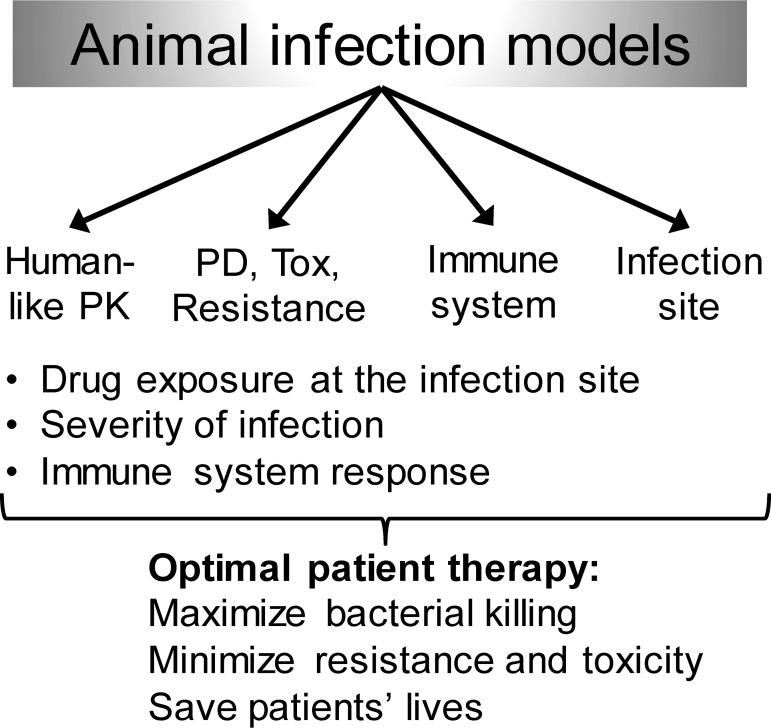
Overview of important variables which contribute to the outcome of animal infection models. These factors may need to be considered for study design and execution as well as for the data analysis and ultimate translation of rationally optimized regimens to patients. Tox, toxicity.

Laboratory animal models have been used for decades to identify effective dosing regimens for clinical trials. Although dosages, drug clearance (including metabolism), and other factors often differ considerably between animals and humans, *in vivo* models play a critical role in characterizing the PK/PD for antibacterial agents (Fig. 3). Animal models provide an *in vivo* infection environment and anatomical barriers that are difficult to reproduce *in vitro*. Animal infection models can forecast drug efficacy in patients, and the probability of regulatory approval increases with the probability of PK/PD target attainment (1, 2, 72, 100).

The most widely used *in vivo* models for antibacterial PK/PD are the murine thigh and lung infection models (100). The thigh model is performed by injecting a bacterial suspension directly into the musculature of one or both thighs. The most commonly used lung infection model is performed by pipetting droplets of a bacterial suspension onto the nares and allowing the mice to inhale the inoculum. Both models often use cyclophosphamide-induced neutropenic mice to allow growth of a range of bacterial pathogens. Some bacterial strains can also produce robust infections in “normal” (i.e., nonneutropenic) mice, which provide information about the contribution of the immune response to the drug efficacy and may be better suited for studying resistance, the latter of which necessitates use of higher inocula. The primary endpoint is reduction of the bacterial burden in the infected tissue, which is typically assessed at 24 or 48 h after initiation of antibiotic therapy. Bacteriostasis, 1- or 2-log_10_ bacterial killing at 24 h (compared to the burden at the time therapy is initiated), is often used as an endpoint and has been shown to correlate with clinical outcome, including in patients with infections such as hospital-acquired pneumonia, community-acquired respiratory tract infections, bacteremia, and complicated skin and skin structure infections (1, 2, 100). Of note, 2-log_10_ bacterial killing in mice at 24 h may not be achievable by slowly killing (bacteriostatic) antibiotics. Considerable amounts of published data are available for many antibacterial agents in mice that can be used as positive controls; this presents a particular advantage of the murine neutropenic thigh and lung models compared to larger-animal models.

## CONSIDERATIONS FOR DESIGN AND CONDUCT OF *IN VIVO* PK/PD MODELS

### Pharmacodynamic studies.

Although the basic approach to conducting *in vivo* PK/PD studies is fairly standard, there is considerable variation among laboratories in the details of study design and conduct. These details can have a large impact on the results and should be carefully considered (101). Recommendations (Table 2) have been developed based on experiments that predicted clinical success (1, 2, 100), and this topic has been reviewed previously (101). Some recommendations may need to be adapted for specific drug-pathogen combinations or for other animal models. Benchmarking studies and the inclusion of comparator active control therapies to establish appropriate experimental conditions can enhance the utility of animal infection models and the robustness of predictions for translation to patients.

**TABLE 2:** Recommendations for murine neutropenic thigh and lung infection models to determine nonclinical *in vivo* PK/PD targets^*a*^

Study component	Recommendation^*b*^	Comments
Mouse strain	Outbred (e.g., CD-1, ICR or Swiss Webster)	Historically female; studies in both sexes have been strongly encouraged recently and, if feasible, should be considered
Induction of neutropenia	Cyclophosphamide i.p. or s.c. at 150 mg/kg of body weight at 4 days prior to infection and 100 mg/kg at 1 day prior to infection	Results in <100 neutrophils/mm^3^ for at least 2 days
Inoculum preparation	Culture should be in log-growth phase	Subculture aliquot from an overnight broth culture in fresh medium for several hours prior to study start
Mouse inoculation	Infect thigh via i.m. injection of 100 μl and lung via intranasal inhalation of 50 μl (i.e., 25 μl per naris)^*c*^	Culture for inoculation should be 10^6^ to 10^7^ CFU/ml
Baseline bacterial burden	10^6^ to 10^7^ CFU/tissue (may differ by pathogen and strain)	Note that this represents the burden at the time therapy begins
Start of therapy	2 h postinfection	Delay may be necessary for baseline tissue burden to reach 10^6^ to 10^7^
Study duration	24 h (sometimes 48 h)	After start of antibacterial dosing
Bacterial growth over study period	Tissue burden should increase by 2–3 log_10_ CFU in untreated mice compared to baseline at initiation of therapy	Note that this assumes that the initial inoculum is sufficiently below the plateau for a given strain; the use of less-virulent strains may result in underestimation of the PK/PD target
No. of strains	At least 4 strains of each target pathogen (including a reference strain), if possible, with relevant resistance profiles and mechanisms	Include enough strains to assess strain-to-strain variability; mean and median PK/PD target values should converge
Bacterial phenotypes	Cover MIC range of compound, include clinically relevant resistant phenotypes	Consider *in vivo* virulence when choosing strains
Control therapies	Inclusion of active comparator control (e.g., standard of care) may be beneficial; dosage regimen (with/without humanizing) should be considered	Especially important for evaluation of combination therapies against multidrug-resistant strains; dosing algorithm should be supported by PK/PD considerations

a Data are from Andes and Lepak (101). CD-1, outbred strain of albino mice; ICR, outbred strain of albino mice; i.p., intraperitoneal; s.c., subcutaneous; i.m., intramuscular.

b These specific recommendations are for “routine” establishment of PK/PD targets. Study design elements may need to be modified to achieve different experimental goals. Examples include the use of other bacterial phenotypes (including growth stages), use of immunocompetent mice (which can inform how targets may differ in the presence of white blood cells and/or support longer treatment durations), and use of a different bacterial burden (such as using a higher burden to study resistance).

c The maximum volume of the bacterial suspension which can be given per naris will depend on the mouse weight. This volume may affect the regional deposition of bacteria in the lung.

Consideration of the number of mice per group is an important design choice for PD studies. It is difficult to provide explicit guidance on the number of animals required to appropriately power a study since it depends on a variety of factors (such as variability associated with a model, strain, or drug; the number of groups within an experiment; and the type of analysis to be conducted). Sample sizes can be calculated for statistical comparisons of viable counts at the end of therapy via *t* test or analysis of variance (ANOVA) statistics (see the supplemental material). As these analyses consider only a single time point, the resulting samples sizes are conservative (i.e., higher) compared to the sample size required for time course analyses via population PK/PD modeling. The latter approach estimates treatment differences based on the time course of viable counts at multiple sampling times.

In practice, there are typically four observations collected for each group using the standard neutropenic thigh or lung infection models, and consideration should be given to studying both sexes. Of interest, when using the thigh model, many investigators utilize the two thighs as independent samples (thus including only 2 mice per group). Although this reduces the overall number of animals required, it may not be a best practice since the two samples from the same animal are not independent. We recommend that the design and conduct of studies be supported by prospective statistical or modeling analyses to ensure that an adequate number of truly independent observations are obtained to appropriately power the experiment for the intended purpose.

### Plasma protein binding.

In order to interact with its molecular target, a drug must be freely available (e.g., not bound to host proteins), and only unbound drug molecules can penetrate through the outer membrane of Gram-negative pathogens. Therefore, results from *in vivo* studies should be adjusted for protein binding and expressed in terms of free (*f*), i.e., non-protein-bound drug. It is recommended to conduct protein binding studies across a relevant concentration range with an appropriate *in vitro* assay. Whenever possible, at least 3 concentrations covering the anticipated *in vivo* plasma and tissue concentrations should be studied. A number of different *in vitro* assays are available. Currently, equilibrium dialysis is considered the reference method and is preferred over ultracentrifugation (102). The most accurate measurements can be made using radiolabeled drug; however, this may not be possible in the early stages of development. Typically, a single protein binding value is determined (for example, an average across the concentrations tested) and all *in vivo* PK measurements are adjusted by multiplying the measured concentration by the assumed free percentage. If significant concentration-dependent binding exists, this nonlinear binding should be incorporated into the data analysis using mathematical modeling.

### Pharmacokinetic studies.

Generating high-quality PK data is critical for PK/PD analyses. The goal of PK experiments is to define the time course of drug concentrations in plasma, serum, or blood and potentially at the primary infection site. Several factors need to be considered for study design. As a best practice, exposure data should be collected from animals under the same conditions as the PD studies since infection may alter the PK (e.g., clearance and volume of distribution). If different matrices are collected across species (e.g., if drug concentrations are measured in whole blood for animal studies but in plasma for human studies), then red blood cell (RBC) partitioning needs to be determined and used to adjust for blood/plasma differences. Characterizing the PK at the infection site becomes comparatively more important for deep infection sites that equilibrate slowly or poorly with plasma and may be sequestered due to the infection (24, 48, 49, 103, 104).

If a drug is being developed for treatment of bacterial pneumonia, it is recommended to utilize lung infection models for both PK and PD and to determine lung epithelial lining fluid (ELF) concentration data. The latter is critical since the drug exposure profile at the infection site may substantially differ from that in plasma. The “gold standard” approach in both clinical and nonclinical studies is to characterize drug concentrations in ELF, which is believed to represent the key compartment for infections by extracellular pathogens. Briefly, bronchoalveolar lavage (BAL) is performed, and the BAL fluid is gently centrifuged to remove alveolar macrophages and other cells; this prevents bias in the ELF concentration, since some drugs accumulate extensively in these cells. Drug concentrations in the supernatant (i.e., diluted ELF) are measured and adjusted for the lavage dilution factor using the urea correction method (48, 105–108). This yields the drug concentration in the ELF. The cell pellet may also be utilized to determine concentrations within alveolar macrophages (105); these intracellular drug concentrations can be particularly important for some drugs (such as macrolides) and infections.

For logistical reasons, systemic and/or tissue PK data are usually obtained separately in satellite PK experiments. A sufficient number of dose levels (usually 3 to 4) are needed to identify and characterize nonlinear PK, if present, and these should include the smallest and largest doses used in the PD studies to minimize extrapolation outside that range. The PK samples are typically collected via terminal procedures; thus, each animal usually contributes one concentration measurement at a single time point (especially in mice). Collecting serial blood samples from the same animal (e.g., multiple retro-orbital, facial vein, or tail vein bleeds) at different time points better informs the PK parameters and allows one to separate animal variability from residual error noise (e.g., bioanalytical noise). Serial blood sampling may not be possible in all infection models; however, methods have been developed and employed by some investigators (109–116). Destructive sampling with one PK sample per mouse remains the most common approach.

Measuring drug concentrations in blood, plasma, and BAL fluid (for ELF) can usually be accomplished via sensitive and specific liquid chromatography-tandem mass spectrometry (LC-MS/MS) assays. These are preferred over older bioanalytical methods (such as bioassays) because of their superior specificity, sensitivity, and precision. Bioactive metabolites should also be measured and accounted for, if they are present at relevant concentrations.

### PK sampling times.

Due to technical limitations and animal welfare considerations, there is a practical limit of approximately 6 to 8 sampling time points during any given experiment. Sampling times should be carefully chosen (and informed by any available PK data) to provide robust information within these experimental constraints. Studies should be designed and repeated, if necessary, to adequately capture information related to the absorption phase, peak concentration, drug distribution, and elimination. Ideally, the chosen sampling times should reasonably characterize the overall drug exposure (i.e., the area under the curve [AUC]), the terminal half-life, and the time when drug concentrations decline below the lowest MIC of interest.

Mathematical modeling and simulation approaches (including optimal design methods) can be prospectively applied to select the most informative sampling time points prior to conducting the PK experiment (117–121). If the design is suboptimal, the study may not provide adequate data to fully characterize the drug exposure profile. This is important because even the most sophisticated retrospective PK modeling and simulation approach will not compensate for poorly informative data; accuracy of PK predictions will suffer and, ultimately, the calculated PK/PD targets may be biased. If no or insufficient prior PK data are available to aid in study design, a small pilot experiment may be warranted. Collection of high-quality PK data may require multiple, sequential experiments. This iterative process is considered best practice if a single experiment does not adequately capture the PK profile. Although this approach may be complicated by factors such as limited time, resources, and drug supply, it is imperative to collect suitably informative PK data.

Studying drug combinations is more complex than evaluating monotherapies and requires additional consideration, such as potential drug-drug or drug-vehicle (e.g., for dimethyl sulfoxide [DMSO]) interactions. Furthermore, it is important to ensure that both drugs combined are present at the primary infection site at the same time. The design and interpretation of combination PK (and PD) studies benefit greatly from prospective application of mathematical modeling and optimal design approaches that are beyond the scope of this review (117–125).

### Testing human-like exposures.

The PK/PD index (e.g., *f*peak/MIC, *f*AUC/MIC, or *fT*>MIC) and its magnitude required for a chosen efficacy endpoint are typically determined using murine infection models. However, drug half-lives are usually much shorter in mice than in humans (126), which results in concentration-time profiles with different shapes, even if the two profiles are matched in the AUC. The importance of this aspect for bridging from animals to humans has been shown by Deziel et al. (127), where different dosage regimens were designed to achieve human-like levofloxacin concentration-time profiles but did not result in equivalent efficacy. Evaluating humanized PK profiles in animals can provide complementary information to traditional PK/PD indices and should be considered during drug development. Additional guidance on humanization (87) is provided in the supplemental material.

### Analysis of PD data.

To analyze viable bacteria count data (e.g., CFU at 24 h) at a single time point, a Hill model is commonly employed. Characterizing exposure-response relationships (e.g., *f*AUC/MIC versus effect) is strongly preferred over dose-response relationships (72), since the former account for PK and are thus much more informative. This basic PD approach is often useful for optimizing antibacterial monotherapy based on single-time-point data. If multiple time points are evaluated (from different mice), population PK/PD modeling can characterize the time course of bacterial killing and regrowth. Empirical, MB, and QSP mathematical PK/PD models can be used to describe and predict the drug effect over time to rationally optimize dosage regimens as described above for *in vitro* models.

### PK modeling approaches.

Drug concentration profiles can be modeled by various approaches (128, 129), depending on the type of experimental data collected, the complexity of the system (e.g., linear versus nonlinear PK), and the skill set of the modeler. For a typical data set that contains one measurement per animal (e.g., terminal sampling at a single time point), naive pooling is often used. For this approach, all observations at a given dose are assumed to come from one animal. Alternatively, naive averaging can be employed by calculating the average concentration at each time point. Both naive approaches ignore between-subject variability and estimate only one pooled value for clearance and one for volume of distribution. Estimates tend to be biased unless variability is low (e.g., coefficients of variation [CV] are less than approximately 15%) (128–130). To obtain standard errors for these data sets, the Bailer method (131, 132) and bootstrap resampling techniques have been developed (133–135). The Bailer method uses linear combinations of mean concentrations at different time points to statistically compare drug exposures between treatment groups. The bootstrap resampling approach randomly creates a number of pseudoprofiles to allow for statistical comparisons and to estimate the between-animal variability; this method is very flexible and uses noncompartmental techniques for analysis of the pseudoprofiles.

If serial samples are obtained from the same animal, the standard two-stage method can be used where the data from each animal are fit separately. If each profile characterizes all PK phases (i.e., absorption, distribution, and elimination), this method provides reasonable estimates of the mean PK parameters, but it may substantially overestimate the variability between subjects (128, 129). Fitting the average plasma concentration profile via naive pooling or the standard two-stage approach may be adequate to predict the mean concentration profile for data sets with low between-subject variability. This allows a broader range of scientists to perform PK modeling and to progress a drug development program efficiently. However, for data sets with high between-subject variability, nonlinear PK, or multiple different types of observations (e.g., plasma, ELF, urine, or efficacy data), population modeling offers substantial benefits.

### Population PK modeling.

Population modeling borrows information across all subjects by fitting one subject in the context of all other subjects. This approach can simultaneously describe and predict drug exposures in multiple compartments, such as plasma and ELF (108, 136–139), and enables Monte Carlo simulations to predict the range of expected exposure profiles in patients (1, 14, 140). Population estimation algorithms have proven robust to estimate PK parameters for both frequently sampled and sparse data sets (130, 137) and are the method of choice for drugs with nonlinear PK and for data sets with sparse sampling. These include data sets with one plasma and ELF concentration per mouse. Population modeling is particularly powerful if advanced estimation algorithms based on the exact log-likelihood are employed. This approach provides unbiased and precise estimates and predictions in a reasonable time frame considering the time for performing the experiments (Table 3) (130, 137, 141). While full Bayesian approaches are appealing and powerful, they require more time (e.g., for sensitivity analyses) and advanced modeling skills (130, 142, 143).

**TABLE 3:** Comparison of PK modeling and simulation approaches in increasing order of complexity from top to bottom

Approach	Between-subject variability	Accuracy of predictions	Comments
Naïve pooling	Ignored (i.e., assumed to be zero or very low)	Only mean profiles can be predicted	Can be adequate to simulate mean concentration profiles, if variability is low; yields biased predictions if variability is moderate or large; cannot simulate between-subject variability
Standard two-stage	Often overestimated	Predicted concn range may be too broad	Can be adequate to simulate mean concentration profiles, if variability is low; requires serial sampling, which may be problematic for mouse PK studies
Population modeling (approximate log-likelihood)	Bias can be large for sparse data	Can simulate variability, but may be considerably biased	Can simulate mean concentration profiles and between-subject variability but may yield biased results for sparse data
Population modeling (exact log-likelihood)	Often most suitable choice	Often most reasonable choice	Can simulate mean concentration profiles and between-subject variability with no (or less) bias; can handle complex PK models with multiple dependent variables (e.g., PK, PD, and resistance)
Population modeling (advanced three-stage methods)	Very powerful, can leverage prior information via a Bayesian approach	Can account for uncertainty as well as for between-subject variability	Powerful, but more complex; requires more expertise and modeling time (e.g., for sensitivity analyses)

## CHALLENGES OF *IN VIVO* STUDY CONDUCT AND INTERPRETATION

The success of characterizing PK/PD in animal models depends largely on sound experimental design, suitable data analysis, and the ability to control variance. This involves learning and refining in an iterative fashion to understand the sources of variability and then to minimize variance until the results converge around a final PK/PD target. This process benefits greatly from being executed by a close-knit, highly functional team that regularly discusses experimental designs, results, and interpretation. Several scenarios warrant special attention.

### Pharmacokinetic considerations:

• Drugs with short half-lives in rodents can complicate study design (e.g., when the goal is to achieve a wide range of exposures in dose fractionation studies).
• Species specific toxicities or PK profiles may hinder the ability to understand the full exposure-response (e.g., when sufficiently high doses to observe near-maximal effect cannot be tested).
• Incorporating tissue concentration data may be complicated, and yet it should not be assumed that the extents and rates of penetration are the same across animal species and humans. For pneumonia, approaches have been established and applied to design optimal dosage regimens based on ELF penetration data (48, 87, 105, 144, 145).
• The time courses of penetration at the target site may not mirror circulating drug concentrations and may differ across species (e.g., for oritavancin [104]). This may be particularly critical when maximizing synergy of drug combinations.
• Plasma protein binding of drugs may differ between animals and humans and between “normal” and critically ill patients (146, 147).

### Pharmacodynamic considerations:

• PD models are acute. Severe (often rapidly lethal) infections are usually required for model stability and minimizing variability but may not mimic the course of infections in humans.
• Different PK/PD target values can be obtained from different models, studies, and bacterial strains, as well as from various infection sites and/or test conditions.
• Some studies and bacterial strains may not perform the same as others, even in well-characterized animal models; between-strain variability is expected and can complicate the establishment of PK/PD targets and, subsequently, human dose predictions.
• Opinions vary on which endpoints should be used to establish PD targets (i.e., stasis versus 1- or 2-log_10_ reduction in CFU or, alternatively, using the doses associated with 50% of maximal effect [ED_50_] or 90% of maximal effect [ED_90_]).
• Different endpoints may be required for various types of infections and patient groups (e.g., for immunocompromised patients or those with more-serious infections such as VABP/HABP).
• A more stringent endpoint such as 2-log_10_ reduction in CFU at 24 h in a mouse infection model may not be achievable for slowly killing antibiotics. Studies with longer treatment durations may be warranted to explore this situation.

### Variability within and between studies.

**FIG 4:**
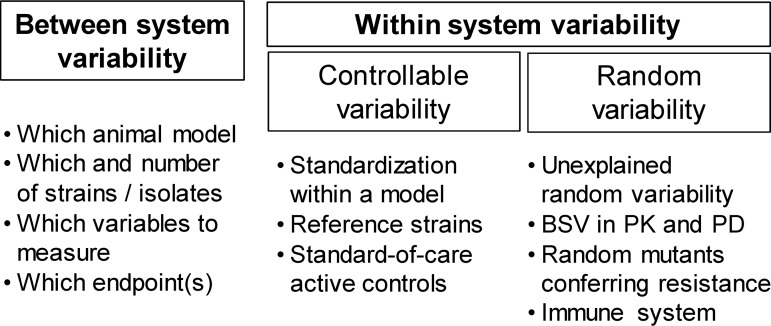
Different sources of variability that may affect the results of animal infection models. The between-system variability can be handled by appropriate choices for and the selection of experiments to be performed. The within-system variability can be split into a controllable portion and a random (i.e., usually noncontrollable) part. Experimental design choices and careful execution of animal infection model studies can minimize the controllable variability. The random, unexplained variability will necessarily include components such as between-subject variability (BSV) in pharmacokinetics, pharmacodynamics, the infection site, and the immune system.

Variability associated with the conduct of animal infection models can be largely minimized via careful planning and execution. However, uncontrollable sources of variability associated with the PK, PD, infection site, and immune response will remain and are difficult to control (Fig. 4). This variability may lead to one or more extreme observations, and it can be tempting to remove such a presumed outlier(s). However, with the exception of *a priori* documented experimental reasons (such as those due to a missed dose), removal of outliers is not appropriate and will likely yield biased conclusions. Performing and presenting the data analysis with and without a “suspected” outlier represents good practice, as is the use of a suitable number of experimental replicates. If a whole experimental group (or entire study) appears to be an “outlier,” then a repeat evaluation is warranted. It is important to understand if such results are reproducible and to investigate why the results differ between replicated groups.

**FIG 5:**
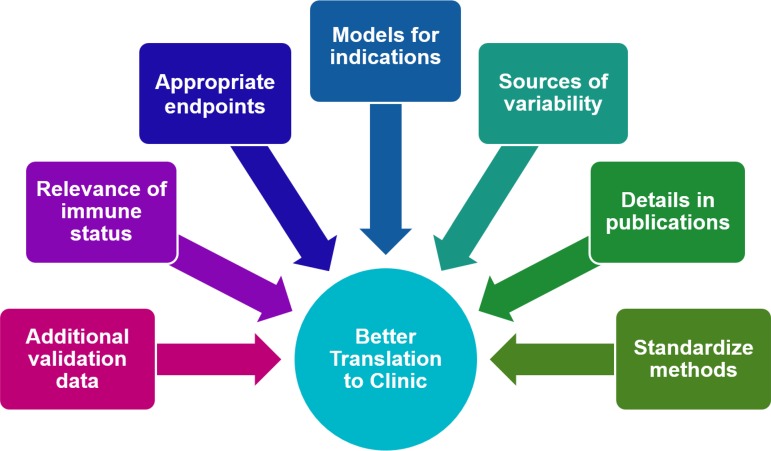
Considerations and perspectives to enhance the robustness of animal infection models and ultimately better translate efficacious and reliable dosage regimens to patients.

It is common for results from studies conducted in different models or by different laboratories to differ to some degree and sometimes widely. In extreme cases, one set of results may support termination of a new drug candidate while another data set for the same compound supports progression. It is likely that differences in the design, conduct, and analysis of studies, even for the “workhorse” murine PK/PD models, contribute to this situation. Careful experiment conduct is critical, and it may be helpful when using the workhorse models to standardize certain components such as inoculum size and preparation, strain fitness, timing of infection, infection site, inoculation method, and immune status. These variables can have a large impact on the results and conclusions (101). It is further helpful to benchmark PK/PD models and methods using relevant positive controls (i.e., effective reference treatments) (Table 2; see also Fig. 5) for which both animal and human PK/PD data are available for the target indication. By use of such active controls, a collection of data under a standardized test methodology can be developed to support drug development and regulatory review. This will allow the performance of a new drug to be assessed in the context of benchmarked controls and endpoints.

### Clinical dose selection.

Guidelines have been published (e.g., by EMA) that recommend calculating PK/PD targets based on specific efficacy endpoints in the workhorse models for different clinical indications (6–8). In general, more antibacterial effect is required for more serious infections. Thus, targets based on no change in viable counts (stasis) or on a 1-log_10_ reduction in CFU compared to pretreatment baseline have been recommended for less-severe infections such as skin and soft tissue as well as for complicated urinary tract infections (cUTI); in contrast, 2-log_10_ reductions in CFU have been suggested for more-severe infections such as pneumonia (43). Importantly, these endpoints are calculated relative to the bacterial density at initiation of antibiotic treatment and not relative to the viable counts of the growth control group at end of therapy. The rationale for a higher 2-log_10_ hurdle is to rapidly reduce the bacterial burden to a density that can be controlled by the immune system; in the latter case, the surviving bacterial population is so small that the risk for emergence of resistance during therapy due to *de novo* formation of resistant mutants is low (1, 14). Although these are laudable goals, focusing on specified endpoints requires standardized model systems with benchmarking based on positive controls. Such highly controlled animal infection models currently do not exist.

Aiming for a stringent target endpoint (e.g., ≥ 2-log_10_ reduction in CFU) or the maximum tolerated dose is common in the early stages of clinical drug development. High doses may help mitigate potential PK concerns, such as low drug exposure at the primary infection site, altered PK in special populations, and substantial variability in patients. However, almost invariably, the amount of drug that can be dosed in patients is limited by nonclinical safety coverage, clinical adverse events, lack of therapeutic index, cost of goods, and other factors. This typically leaves two options. First, drug developers can keep the same target endpoint and risk not covering the encountered MIC range; second, a less stringent endpoint (e.g., stasis or 1-log_10_ reduction instead of 2-log_10_) could be used to set the target. The latter choice is the more common path, as not being able to cover the full MIC range is a poor starting point for a new drug and creates problems for establishing susceptibility breakpoints. However, use of less-stringent endpoints may reduce the probability of achieving an adequate therapeutic response for more-severe infections, can accelerate the development of resistance, and may result in breakpoints that are higher than appropriate. In this scenario, characterizing the impact of the immune system and, if mutants with reduced susceptibility are found, assessing their fitness in animals as well as evaluating combination therapies for severe infections may be a path forward.

Despite these complexities, the guiding principle should always be the scientific method, and there are steps that can provide additional confidence in the chosen nonclinical PK/PD targets and endpoints. It is best practice to generate data in more than one model system (i.e., another animal model and/or dynamic *in vitro* models). To enhance the information gained from the primary endpoint (e.g., reduction in CFU), secondary endpoints such as analyses of viable counts of resistant bacteria, biomarkers, survival, histopathology, inflammatory markers, radiology, bioluminescence, and others can provide valuable insights. Concerns may arise if discordant results are obtained from different model systems and bacterial strains. However, this should not dissuade drug developers from conducting different types of experiments. Discordant results can be actively managed and explanations for the differences sought, and the insights gained can be highly valuable.

### Future perspectives on *in vivo* models.

The field of antibacterial pharmacology is fortunate to have a considerable armamentarium of PK/PD tools and expertise. Commonly used models (such as murine neutropenic thigh and lung models) have provided a sound basis to date. However, PK/PD is an evolving discipline, and challenges as well as open questions remain. The practice of optimizing, standardizing, and benchmarking the workhorse models likely ensures better reproducibility from study to study and from laboratory to laboratory and enhances our ability to interpret the results for different types of infections and various antibacterial classes (Fig. 5). Leveraging suitable modeling, simulation, and optimal design approaches and engaging team members across disciplines to discuss feasible study designs, results, and clinical goals are undoubtedly highly mutually fruitful.

Establishing additional animal models for PK/PD characterization would expand the translational tools available to the community. The murine thigh infection model reasonably mimics soft tissue infections, and the mouse lung infection model mirrors pneumonia. However, neither may be ideal for characterization of PK/PD profiles at other infection sites. For lower urinary tract infections (e.g., cystitis), urine and/or bladder wall concentrations are likely important for efficacy. However, the mouse thigh model may not be adequate to determine reliable PK/PD targets for these infections, and other validated models do not (yet) exist. Similarly, there is a need for better models to characterize PK/PD for complicated intra-abdominal infections (cIAI) and cUTI, especially since these are common target indications for phase II studies. A rat model for cIAI is available (148, 149); however, some laboratories may not be able to conduct this model due to the increased complexity and animal species. As a surrogate, the neutropenic murine thigh infection model can be a reasonable alternative for infections involving a rapidly equilibrating PK compartment such as pyelonephritis, where intrakidney concentrations are important; however, more data are required to fully assess nonclinical-to-clinical translation in these instances. Consideration should also be given to the development of models that better mimic human disease (e.g., more-natural disease progression), although such models are likely to be low throughput and less practical for routine PK/PD characterization. As one example, rabbit infection models have been developed and can provide serial blood samples for assessing PK and biomarkers of efficacy and safety over time (150–152). In combination with results from murine infection models, these more complex models could provide supporting information for new drugs and play an increasingly important role during drug development.

A final point for consideration is publication of PK/PD data. It is important to provide sufficiently detailed information to allow readers to assess the validity of the work and resulting PK/PD targets and to reproduce the methods employed. All pertinent details of the experiments (including detailed experimental protocols) and associated data analyses (including units, modeling choices, and the enabling equations of the final model) should be published, at least in the supplemental material. For common models and analyses, workshops with hands-on example data sets and (video) tutorials can provide effective training tools. Variability in PD response should be reported and details on the performance of individual bacterial strains (e.g., growth in untreated control animals and variability of drug effect) and their individual PD targets provided. The PK data should be adequately described and a thorough assessment of the quality of the modeling and simulation methods provided (including an evaluation of bias and precision). It is suggested that editors consider the ARRIVE guidelines (153) to ensure adequate reporting of *in vivo* data and an extended set of criteria specifically for PK/PD studies to improve the quality of publications. Collections of resistant bacterial strains (e.g., from CDC and ATCC) are available, and future research and joint discussions are needed to select suitable reference strains.

## CONCLUSIONS

Both *in vitro* and *in vivo* infection models provide powerful PK/PD information and have been shown to predict clinical outcomes. This review provides perspectives on current models, applications, challenges, potential issues, and paths forward. This is a healthy and required evolutionary process to define and critique available methods. The goal is to improve approaches, models, study designs, study performance, analyses, interpretation, and communication. Optimizing the available translational PK/PD tools has become increasingly important as we rely more and more on nonclinical data to predict successful clinical treatment regimens, often to combat serious infections by multidrug-resistant bacterial “superbugs.”

Guidelines for conducting and interpreting nonclinical models are meant to improve the process, not to stifle innovation or eliminate the need for rational thought. Regular discussions among multidisciplinary project teams are essential to optimally leverage these translational tools, and early/frequent discussions with regulatory agencies are critical to maximize utility of the data. Future studies will likely identify scenarios where the recommendations in this review will need to be modified for special infection models, bacterial strains, innovative combination regimens, and novel-acting therapies. Some therapies may require special considerations, and PK/PD approaches should be tailored to the specific needs of the individual compound or drug class and ultimately to the target patient population.

## Supplementary Material

Supplemental file 1
