# Supplementary material for: Generating Robust and Informative Nonclinical In Vitro and In Vivo Bacterial Infection Model Efficacy Data To Support Translation to Humans
Source: Antimicrob Agents Chemother. 2019 Apr 25;63(5):e02307-18. doi: 10.1128/AAC.02307-18 (PMC6496039; doi:10.1128/AAC.02307-18)
Supplement: Supplemental file 1 [file AAC.02307-18-s0001.pdf]

SUPPLEMENTAL MATERIAL

Useful formulas

Total initial inoculum (CFU) = initial inoculum (CFU/mL) ● broth volume of infection site

Expected number of resistant mutants before treatment (Emut) = Total initial inoculum ● MF

with MF being the mutation frequency on normal (i.e. non-logarithmic scale). This number is typically between approximately 10^{-6} and 10^{-9} and has no unit.

EXCEL formula for likelihood of having at least one resistant mutant in one arm:

Prob(at least 1 mutant present) = Poisson(0, Emut, False)

Note that the False indicates a non-cumulative distribution.

Formula for likelihood of having at least one resistant mutant in ALL n arms:

Prob(at least 1 mutant present in all n arms) = [Prob(at least 1 mutant present) $]^n$

Additional best practices for static in vitro systems.

Adequate oxygenation of the bacteria in the SCTK model should be ensured by allowing an appropriate broth to air ratio and by vigorous shaking of the cultures. Most investigators use 50 mL conical tubes. These typically contain 10 to 20 mL of broth to provide a reasonable broth to air ratio. Larger volumes can be evaluated using Erlenmeyer flasks in an orbital shaker where the broth volume should ideally not exceed approximately 15% of the entire Erlenmeyer flask volume for optimal oxygenation.

If drug stability requires supplementary doses to be added during the experiment, the amount and frequency of such doses should be informed by drug stability data in

broth. Furthermore, care must be taken to monitor potential depletion of nutrients or oxygen, changes in medium pH, and accumulation of bacterial waste products during static time-kill experiments. If present, these issues may substantially alter antibiotic potency and accelerate drug degradation, as well as change bacterial growth, metabolic state and the expression of bacterial resistance mechanisms.

Using in vitro models to study emergence of resistance.

To suppress the emergence of high-level resistance, determining the antibiotic dosage regimen that kills the pre-existing less-susceptible bacterial population (i.e. first-step mutants) can be highly beneficial. This greatly minimizes the likelihood that second-step mutation(s) and emergence of high-level resistance due to additional resistance mechanism(s) occur during therapy. This has been described in the literature for levofloxacin, where suppression of mutants overexpressing efflux pumps prevented amplification of target-based mutants (1). In immunocompetent patients, the immune system may contribute to killing less-susceptible mutants. To maximally exploit this, rapid killing of the predominant susceptible population is essential, since the immune system is saturated at a certain bacterial density (2, 3).

Designing human-like exposure profiles in animals.

Bridging antibiotic exposures from animals to humans has generally been performed using only the relevant PK/PD index (4). The example of levofloxacin shows, however, that the partially humanized PK profiles with dosing every 12 h (**Figure S1**, panel A) or every 8 h (**Figure S1**, panel B) differed in the time-course of antibacterial

effect (**Figure S1**, panel C). Therefore, it is important to understand how the shape of the concentration-time profile and frequency of dosing affects efficacy as well as the PK/PD index. Human-like concentration-time profile(s) can be predicted via population PK modeling and Monte Carlo simulations. For example, concentration-time profiles for the 2.5^{th} and 97.5^{th} percentiles (which characterize the 95% prediction interval of concentrations expected in patients) can be predicted. Dosages for lab animals can then be defined to provide concentration-time profiles within these limits.

For antibiotics with short half-lives in small animals, such as β-lactams, maintaining drug concentrations within the 2.5^{th} and 97.5^{th} human percentiles can be difficult and not feasible, unless computerized infusion pumps are employed. Ethical and logistical considerations place limits on the frequency of dosing; therefore, to maintain concentrations above a certain level, mice usually receive a relatively high dose as frequently as possible. These humanized concentration-time profiles in animals typically have higher peak concentrations and shorter times above a threshold concentration (e.g. the MIC) compared to those in humans. This limitation can be mitigated by using programmable infusion pumps to administer a controlled profile to cannulated animals (5-9); however, this advanced technique is more resource and cost intensive and not yet widely available.

Translational PK/PD modeling can support a rational choice of humanized dosage regimens for animals; these regimens can be optimized via mechanism-based or Quantitative and Systems Pharmacology modeling (see main text) to match the drug concentrations and receptor occupancy profiles in humans within the logistical constraints. Ultimately, both efficacy and safety considerations (due to concentrations in

animals declining below the 2.5th or increasing above the 97.5th percentile, respectively) will need to be acknowledged. Active discussion among team members with cross-discipline expertise can be helpful to optimize the humanization of PK in mice (within the non-negotiable limitations) to improve the design of nonclinical infection model studies and support more robust translation to the clinic.

Additional *in vivo* PK considerations.

The impact of protein binding on the PK/PD index is significant because the free fraction of drug is typically considered to be pharmacologically active. Binding saturation may result in exaggerated estimates of free drug fraction at high concentrations, and atypical binding kinetics have implications on dose escalation (10). Protein binding may result in over-inflation of the PK/PD index when the drug exposure is very low. This situation may occur for highly bound drugs (i.e. binding of ~99%) which have a larger uncertainty in the target value of the PK/PD index. If present, enterohepatic recirculation may greatly prolong the terminal half-lives and increase drug exposures (i.e. area under the curve) both in animals and patients, and may thus need to be considered when scaling from animals to humans (11).

Power calculations.

Before conducting an animal infection model study, it is important to calculate the power to detect a relevant difference in the $\log_{10}$ viable counts between a treatment and control arm. The sample size calculations provided below assume that $\log_{10}$-transformed viable count data are to be analyzed by an unpaired t-test using a significance level

(alpha) of 0.05. **Table S1** applies to a study design with 4 animals per time-point for each treatment or control group. A low standard deviation (i.e. residual error) of 0.4 to 0.6 on a log_{10} scale may be achieved by experienced experimental teams under controlled conditions for the total and resistant bacterial populations. However, larger standard deviations of 1 to 1.4 may be encountered, especially when the resistant population is quantified. Only the smallest standard deviation yields a power above 80% for detecting a 1.0 log_{10} difference. For large standard deviations (i.e. 1.2 or 1.4 on log_{10} scale), more than 4 animals per time-point are required, since the power to detect a significant difference is considerably below 80% for most effect sizes. If multiple comparisons are to be performed, an alpha-adjustment would need to be considered.

Viable count data at multiple time-points can be fit simultaneously via empirical, mechanism-based, and Quantitative and Systems Pharmacology (QSP) models using population estimation methodology. These time-course modeling approaches yield a higher (i.e. better) power to detect significant differences between groups compared to those in **Table S1**. Moreover, mechanism-based and QSP modeling can simultaneously fit the time-course of viable bacterial counts for the total and antibiotic-resistant bacterial populations to further enhance the robustness and power of the analysis.

Table S1: Power to detect a 1.0, 1.5, 2.0, 2.5 or 3.0 log_{10} difference of the log_{10} viable counts between two treatment or control groups via an unpaired t-test at a significance level of 0.05 using n=4 per group. The table shows low (0.4 to 0.6), intermediate (0.8 to 1.0) and high (1.2 to 1.4) standard deviations for the residual error on a log_{10} scale.

**Standard deviation on log_{10} scale**	**Effect size** (i.e. difference between groups)				
	1.0 log_{10}	1.5 log_{10}	2.0 log_{10}	2.5 log_{10}	3.0 log_{10}
0.4	84%	99%	>99.9%	>99.9%	>99.9%
0.6	51%	84%	97%	99.8%	>99.9%
0.8	32%	60%	84%	95%	99.2%
1.0	22%	43%	66%	84%	94%
1.2	17%	32%	51%	69%	84%
1.4	14%	25%	40%	56%	72%

Figure S1: Observed (markers) and model fitted (continuous lines) plasma concentrations (panels A and B) and efficacy (panel C) of levofloxacin against *Bacillus anthracis* under “partially humanized” animal pharmacokinetic profiles. Panels A and B show treatment regimens in which levofloxacin was dosed at the beginning of each 24-h dosing interval (AUC = 23 mg · h/liter) with a smaller dose at 12 h (AUC = 6.1 mg · h/liter; partially humanized [A]) or in which levofloxacin was given in three decreasing doses at 8-h intervals (AUCs = 22, 7.5, and 4.5 mg · h/liter, respectively; partially humanized [B]). The broken line shows the human exposure (AUC_{24} = 36 mg · h/liter; equivalent to AUC_{24}/MIC = 300). Panel C shows the PD effect of the human exposure profiles and of the “partially humanized” animal exposure profiles against *B. anthracis*. Adapted from Deziel M *et al.* Antimicrob Agents Chemother. 2005; 49: 5099-106.

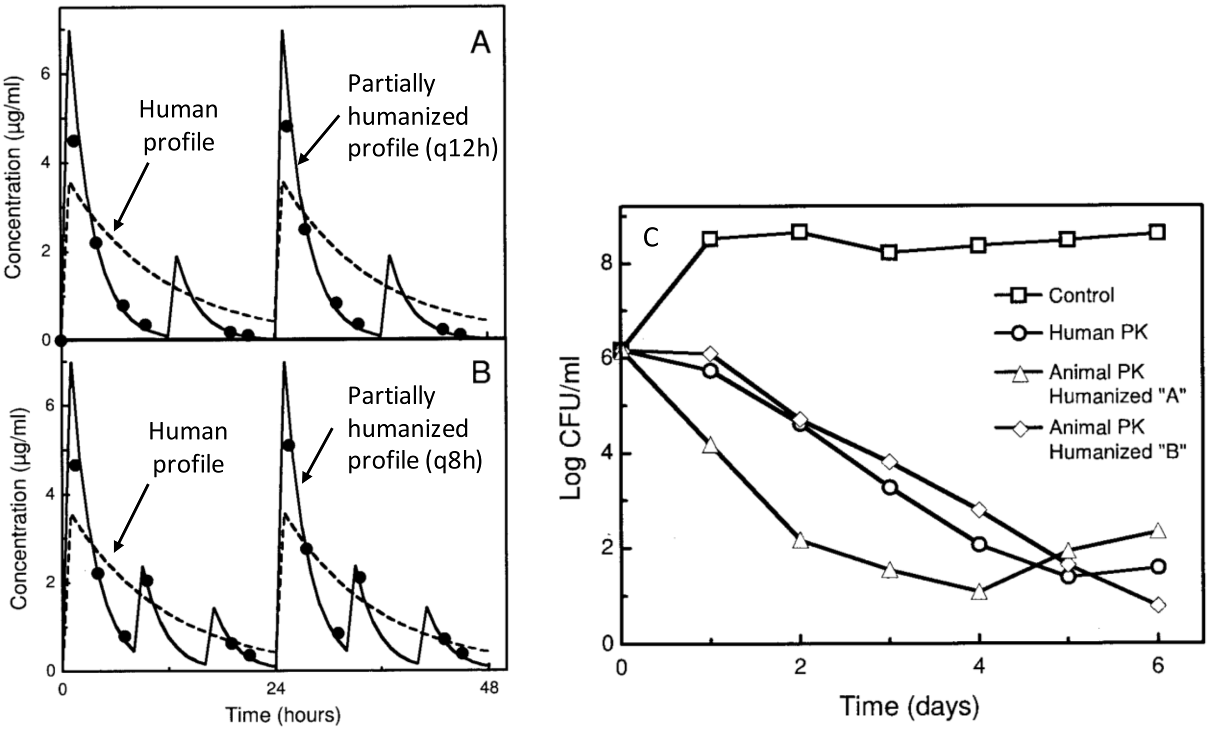

References

1. **Louie A, Brown DL, Liu W, Kulawy RW, Deziel MR, Drusano GL.** 2007. In vitro infection model characterizing the effect of efflux pump inhibition on prevention of resistance to levofloxacin and ciprofloxacin in Streptococcus pneumoniae. Antimicrob Agents Chemother **51:**3988-4000.
2. **Drusano GL, Liu W, Kulawy R, Louie A.** 2011. Impact of granulocytes on the antimicrobial effect of tedizolid in a mouse thigh infection model. Antimicrob Agents Chemother **55:**5300-5305.
3. **Drusano GL, Fregeau C, Liu W, Brown DL, Louie A.** 2010. Impact of burden on granulocyte clearance of bacteria in a mouse thigh infection model. Antimicrob Agents Chemother **54:**4368-4372.
4. **Deziel MR, Heine H, Louie A, Kao M, Byrne WR, Basset J, Miller L, Bush K, Kelly M, Drusano GL.** 2005. Effective antimicrobial regimens for use in humans for therapy of Bacillus anthracis infections and postexposure prophylaxis. Antimicrob Agents Chemother **49:**5099-5106.
5. **Berry V, Hoover J, Singley C, Woodnutt G.** 2005. Comparative bacteriological efficacy of pharmacokinetically enhanced amoxicillin-clavulanate against Streptococcus pneumoniae with elevated amoxicillin MICs and Haemophilus influenzae. Antimicrob Agents Chemother **49:**908-915.
6. **Hoover JL, Singley CM, Elefante P, DeMarsh P, Zalacain M, Rittenhouse S.** 2017. Reducing Antibacterial Development Risk for GSK1322322 by Exploring Potential Human Dose Regimens in Nonclinical Efficacy Studies Using Immunocompetent Rats. Antimicrob Agents Chemother **61**.
7. **Matsumoto S, Singley CM, Hoover J, Nakamura R, Echols R, Rittenhouse S, Tsuji M, Yamano Y.** 2017. Efficacy of Cefiderocol against Carbapenem-Resistant Gram-Negative Bacilli in Immunocompetent-Rat Respiratory Tract Infection Models Recreating Human Plasma Pharmacokinetics. Antimicrob Agents Chemother **61**.
8. **Woodnutt G, Berry V, Mizen L.** 1992. Simulation of human serum pharmacokinetics of cefazolin, piperacillin, and BRL 42715 in rats and efficacy against experimental intraperitoneal infections. Antimicrob Agents Chemother **36:**1427-1431.
9. **Woodnutt G, Catherall EJ, Kernutt I, Mizen L.** 1988. Temocillin efficacy in experimental Klebsiella pneumoniae meningitis after infusion into rabbit plasma to simulate antibiotic concentrations in human serum. Antimicrob Agents Chemother **32:**1705-1709.
10. **Zhou J, Tran BT, Tam VH.** 2017. The complexity of minocycline serum protein binding. J Antimicrob Chemother **72:**1632-1634.
11. **Kim TH, Shin S, Landersdorfer CB, Chi YH, Paik SH, Myung J, Yadav R, Horkovics-Kovats S, Bulitta JB, Shin BS.** 2015. Population Pharmacokinetic Modeling of the Enterohepatic Recirculation of Fimasartan in Rats, Dogs, and Humans. AAPS J **17:**1210-1223.
